# Phenotypic heterogeneity and genetic mechanisms of phage resistance in hypervirulent carbapenem-resistant *Klebsiella pneumoniae*

**DOI:** 10.1080/21505594.2026.2732318

**Published:** 2026-09-09

**Authors:** Ye Huang, Yichen Liu, Shimin Chen, Ziyue Fan, Lili Yan, Fanglin Zheng, Dandan Wei, Yang Liu, Xinping Xu

**Affiliations:** a Jiangxi Provincial Key Laboratory of Respiratory Diseases, Jiangxi Institute of Respiratory Diseases, The Department of Respiratory and Critical Care Medicine, The First Affiliated Hospital, Jiangxi Medical College, Nanchang University, Nanchang, Jiangxi, P.R. China; b China-Japan Friendship Jiangxi Hospital, National Regional Center for Respiratory Medicine, Nanchang, Jiangxi, P.R. China; c Gerontology Department of the First Affiliated Hospital, Jiangxi Medical College, Nanchang University, Nanchang, Jiangxi, P.R. China; d Queen Mary College, Nanchang University, Nanchang, Jiangxi, P.R. China; e Department of Clinical Laboratory, The First Affiliated Hospital, Jiangxi Medical College, Nanchang University, Nanchang, Jiangxi, P.R China

**Keywords:** Phage resistance, CR-hvKP, phage cocktails, *wcaJ* mutation, fitness cost

## Abstract

Phage resistance represents a major obstacle to the clinical application of phage therapy for multidrug-resistant bacterial pathogens. A comprehensive understanding of the phenotypic heterogeneity and molecular mechanisms underlying phage resistance is critical for deciphering bacterium-phage interactions and optimizing therapeutic strategies. In this study, we identified four distinct resistance phenotypes in K2-serotype hypervirulent carbapenem-resistant *Klebsiella pneumoniae* (CR-hvKP) under the selective pressure of phage Kpph1. These resistant strains exhibited either complete or partial resistance, variable fitness costs, and diverse genetic mutations, suggesting multifaceted resistance strategies, including receptor masking, growth inhibition, and community-level resistance. Among these, *wcaJ* mutations were identified as the predominant resistance mechanism, arising from base insertions and integrations of mobile genetic elements, which serve as an efficient pre-adaptive strategy enabling CR-hvKP to evade phage infection. To circumvent this resistance, we isolated a secondary phage, K2V3, which specifically targets *wcaJ*-mutant strains. The rationally designed phage cocktail comprising Kpph1 and K2V3 effectively suppressed all resistant variants and prevented the emergence of further resistance. Collectively, these findings deepen the understanding of phage-bacteria interaction dynamics and provide a crucial theoretical foundation for optimizing clinical phage therapy against multidrug-resistant pathogens.

## Introduction

*Klebsiella pneumoniae* is a major cause of healthcare-associated infections globally. Recently, the pathogen’s ability to acquire exogenous genetic material has given rise to strains exhibiting both hypervirulence and resistance to carbapenem antibiotics [1,2]. This convergence of traits presents a significant challenge to public health [3,4]. Consequently, the World Health Organization (WHO) has designated CR-hvKP as a “Critical” priority pathogen, highlighting the severe threat it poses to human health [5].

Bacteriophages (phages), viruses that naturally infect and kill bacteria, have reemerged as a promising alternative for the treatment of multidrug-resistant infections [6,7]. Unlike conventional antibiotics, phages offer distinct advantages, including high specificity, effectiveness against drug-resistant strains, the capacity to disrupt biofilms, and a favorable safety profile [8]. Despite their therapeutic promise, the rapid development of bacterial resistance to phages remains a major obstacle to their clinical use [9]. Resistant mutants can arise within hours in laboratory experiments [10], a trend that has also been observed in clinical cases [11,12]. By enabling bacteria to evade infection, these resistant mutants may significantly compromise the effectiveness of phage therapy [13].

Bacteria utilize a variety of strategies to defend against phage infection, including blocking phage attachment, preventing entry of phage DNA or RNA, degrading viral genetic material, and inhibiting the release of new viral particles [14–16]. In *K. pneumoniae*, resistance most commonly arises from alterations to surface structures that prevent phages from attaching to the cell [7,17]. The primary target for these mutations is the capsular polysaccharide (CPS), a surface layer heavily involved in the initial phage binding process [18]. Resistance can also occur through modifications to lipopolysaccharides (LPS) and outer-membrane proteins (OMPs), which serve as adsorption receptors for specific phages [19–21]. While numerous studies have elucidated the mechanisms of phage resistance mediate by single or multiple gene mutations, the distribution of these mutations within populations remains unclear [17]. Recent work has further revealed that *K. pneumoniae* employs additional resistance strategies, such as biofilms formation and metabolic inhibition [22,23], suggesting that phage resistance in clinical settings likely arises from a complex interplay of multiple mechanisms. Consequently, the current understanding of phage resistance in CR-hvKP remains incomplete, particularly with respect to strategies for overcoming it during phage therapy.

To elucidate the phage resistance mechanisms of CR-hvKP and devise corresponding therapeutic strategies, we isolated and characterized phage-resistant strains by exposing the K2-type CR-hvKP isolate, NUHL30457, to its specific phage, Kpph1. We identified the underlying genetic mutations and evaluated their impact on bacterial fitness. Our analysis revealed the dominant resistance mutations within the population and identified strains exhibiting concomitant phage-antibiotic resistance. Based on these findings, we developed an optimized therapeutic strategy consisting of a phage cocktail that combined the parental phage with phages targeting the resistant strains. Together, these results highlight the complexity of CR-hvKP defense mechanisms and provide a framework for designing targeted phage cocktails to overcome resistance.

## Materials and methods

### Isolation of phage-resistant mutant strains

The K2-type CR-hvKP strain NUHL30457 was previously isolated [24,25]. The study and consent procedures were approved by the Ethical Committee of the First Affiliated Hospital of Nanchang University (FAHNU), in compliance with the principles of the Declaration of Helsinki (IIT2025369). Prior to data collection, verbal informed consent was obtained from all participants. Because the sampling procedure was noninvasive and posed negligible risk to participants (e.g. burn wound swabbing), verbal consent was used instead of written consent. Phage-resistant mutants were isolated from NUHL30457 using its specific phage, Kpph1 (genus *Webervirus*), previously identified in earlier work [25]. To select for resistance, 100 µL of an exponentially growing bacterial culture was mixed with 100 µL of phage Kpph1 stock at a multiplicity of infection (MOI) of 1. This mixture was combined with 3.5 mL of molten semi-solid LB agar (52°C) and poured onto LB agar plates (Solarbio, L1010). Following incubation at 37°C, bacterial colonies appearing within the centers of the plaques were picked as candidate phage-resistant variants. A single colony from each candidate was isolated using the streak plate method and subjected to three rounds of purification to ensure clonality. Resistance was confirmed using host killing curve assays, spot testing, and efficiency of plating (EOP) assays, as previously described [25,26]. To assess the stability of the resistance phenotype, the isolates were subjected to 10 successive passages on LB agar without phage selection pressure. All confirmed mutants were stored at −80°C in LB broth supplemented with 20% glycerol.

### Host killing curve measurement

The bactericidal activity of phage Kpph1, both individually and in combination with phage K2V3, was evaluated against the parental strain NUHL30457 and its phage-resistant mutants. Infections were carried out at an MOI of 100, with an initial bacterial inoculum of approximately 10^7^ CFU/mL. Bacterial growth was monitored by measuring OD_600_ at 30-minute intervals over a 24-hour period using a Bioscreen C analyzer (Oy Growth Curves Ab Ltd, BP-2200–1). Cultures incubated in the absence of phages served as negative controls, while sterile LB medium was used as a blank to establish a baseline for the instrument. All experiments were performed in triplicate to ensure statistical reliability. The long-term co-evolution experimental conditions aligned with those of the host killing curve, using MOIs of 100, 10, and 1 over a period of 96 hours.

### Phage isolation, characterization, and genome sequencing

Phage-resistant mutants were targeted through the isolation, purification, and characterization of phages, following previously established protocols [25]. Phages were detected using a double agar overlay plaque assay, in which 100 μL of filtered sewage samples was combined with 100 μL of bacterial host culture (OD_600_ = 0.3) and 4 mL of molten soft agar (0.7% w/v agar). This mixture was poured onto solidified 1.5% LB agar plates and incubated overnight at 37°C. Individual plaques were isolated and subjected to three rounds of purification via the double agar overlay assay to ensure uniformity. Purified phages were then amplified by mixing 500 µL of phage suspension with 50 mL of bacterial host culture at an MOI of 0.001 and incubating at 37°C for 5 h. DNA was extracted using the MiniBEST Viral RNA/DNA Extraction Kit Ver. 5.0 (TaKaRa, 9766). Extracted DNA was submitted to Meige Biotech Co. for whole-genome sequencing.

### Phage adsorption efficiency testing

Adsorption efficiency was evaluated to determine the ability of phage Kpph1 to infect the resistant mutants. Briefly, 500 µL of bacterial culture (OD_600_ = 0.5) was mixed with 500 µL of phage suspension at an MOI of 0.001. Following incubation at 37°C for 5 min, the mixture was centrifuged at 12,000 × g for 5 min to pellet bacterial cells. The supernatant was subsequently filtered, and the phage titer was determined using the double-layer agar method. Adsorption efficiency was calculated using the formula: ((initial titer – unadsorbed titer) / initial titer) × 100%.

### Growth kinetics of phage on resistant mutants

The growth kinetics of phage on resistant mutants were assessed as previously described with minor modifications [25]. Briefly, the host bacterium was cultured at 37°C until reaching an OD_600_ of 0.3. A phage-bacterial mixture was prepared by combining 9.9 mL of the host culture with 100 μL of phage suspension to achieve an MOI of 0.01. This mixture was incubated at 37°C for 5 min and then centrifuged at 12,000 × g for 2 min. The resulting pellet was washed and resuspended in fresh LB medium. To initiate the experiment, a 1:100 dilution was performed by transferring 100 μL of the resuspended mixture into 9.9 mL of prewarmed LB medium in flask A. A subsequent 1:10 dilution was made by transferring 1 mL from flask A into 9 mL of prewarmed LB medium in flask B. After 20 and 40 min, 100 μL aliquots were collected from flask B, mixed with 100 μL of host culture, and embedded in 4 mL of semi-solid agar. The agar was overlaid onto LB plates, which were incubated overnight at 37°C. Plaque-forming units (PFUs) were then enumerated to determine phage titers. All experiments were performed in triplicate to ensure reproducibility.

### Scanning electron microscopy

Scanning electron microscopy (SEM) was utilized to evaluate the morphological changes in the phage-resistant mutants. For each strain, 1 mL of Bacterial cultures were harvested during the exponential phase by centrifugation at 6,000 × rpm for 3 min and subsequently washed three times with 1 mL 1× phosphate-buffered saline (PBS) (Life-ilab, AC08L011). The resulting cell pellets were fixed overnight at 4°C in a 2.5% glutaraldehyde solution (Servicebio, G1102-100 ML). Following fixation, samples were rinsed three times with 1 mL ddH_2_O for 6 min each and then dehydrated through a graded ethanol (Xilong Scientific, 64–17-5) series (30%, 50%, 70%, 85%, 95%, and 100%) for 15 min at each concentration. The dehydrated samples were subjected to critical-point drying, sputter-coated with gold for 30 s, and visualized using a GeminiSEM 300 scanning electron microscope.

### Growth curve measurement

The growth kinetics of the phage-resistant mutants were assessed by monitoring optical density at 600 nm. Bacterial cultures in the exponential growth phase were diluted to an initial OD_600_ of 0.01 in sterile LB medium. Growth was measured at 20-minute intervals over 24 h using a Bioscreen C analyzer, with sterile LB medium serving as the blank. All measurements were performed in triplicate.

### Mucus viscosity assay

Capsule-associated mucoviscosity of the phage-resistant mutants was evaluated using a sedimentation assay. Overnight cultures were diluted 1:100 in fresh medium and incubated at 37°C for 6 h. The resulting cultures were normalized to an OD_600_ of 1.0 and centrifuged at 1,000 × g for 5 minutes. The supernatant was carefully collected, and its absorbance was measured at 570 nm to determine mucoviscosity.

### Capsular polysaccharides quantification

Capsular polysaccharides were characterized and quantified as previously described [27]. Phage-resistant mutants were cultured overnight in low-salt LB medium (10 g/L Tryptone (OXOID, LP0042B), 5 g/L Yeast Extract (OXOID, LP0021B), and 0.5 g/L NaCl (Xilong Scientific, 7647–14-5) at 37°C with shaking at 220 rpm, and cultures were normalized based on absorbance at 600 nm. Subsequently, 250 μL of culture was mixed with 50 μL of 1% Zwittergent 3–14 (Aladdin, M105324-5 g) in 100 mM citric acid (Aladdin, C639638-500 g) and incubated at 50°C for 20 min. After centrifugation at 17,000 × g for 5 min, 100 μL of supernatant was transferred to a sterile tube containing 400 μL of ice-cold absolute ethanol and incubated on ice for at least 20 min. The precipitate was collected by centrifugation (17,000 × g, 4°C, 5 min), air-dried for 10 min, and dissolved in 200 μL of ddH_2_O. The polysaccharide concentration was determined using a colorimetric assay. Briefly, 1.2 mL of sodium tetraborate-sulfuric acid (Aladdin, S102367-5 g) solution was added to the dissolved sample, mixed thoroughly, heated in a boiling water bath for 5 min, and immediately cooled on ice for 5 min. The absorbance at 520 nm was measured before and after the addition of 10 μL of 3 mg/mL phenylphenol (Aladdin, P114098-100 mg). The change in absorbance (ΔA_520_) was used to calculate the capsular polysaccharide content.

### Biofilm formation assay

Biofilm formation was assessed using a previously established method with minor modifications [28]. Overnight bacterial cultures were diluted in fresh Tryptic Soy Broth (TSB) (Hopebio, HB4114-19) to a final concentration of 10^6^ CFU/mL. A 125 μL aliquot of each diluted culture was transferred to a sterile, polystyrene 96-well plate and incubated statically at 37°C for 48 h. Wells containing sterile TSB medium served as the blank control. To quantify biofilm biomass, planktonic cells were carefully removed, and adherent biofilms were rinsed three times with PBS buffer and air-dried. The biofilms were then stained with 150 μL of 0.1% (w/v) crystal violet (Servicebio, G1014-50 ML) for 15 min, followed by three washes with ddH_2_O until the control wells appeared colorless. Finally, bound dye was solubilized in 150 μL of 95% (v/v) ethanol, and absorbance was measured at 590 nm using a microplate reader.

### Phage adsorption assay

Overnight cultures of KpR24 were diluted 1:100 in fresh LB broth and grown to an OD_600_ of approximately 0.5. Cells were harvested by centrifugation, washed twice with PBS buffer, and resuspended to approximately 10^8^ CFU / mL. Phage K2V3 was added at an MOI of 0.01, and the mixture was incubated at 37°C for 10 min and 20 min without shaking. The mixture was then centrifuged, and the cell pellet was washed twice with PBS buffer to remove unadsorbed phages. The washed cell pellet was resuspended in LB broth containing 5% (vol/vol) chloroform and vortexed briefly to lyse the cells and release adsorbed phages. After centrifugation to remove cell debris, the released phages in the supernatant were titrated by the double-layer agar method.

For proteinase K treatment, bacterial cells prepared as described above were incubated with proteinase K (0.2 mg/mL) at 37°C for 3 h, and cells were washed twice with PBS buffer before the adsorption assay. A control without proteinase K addition was included.

For LPS competition assays, LPS was extracted from strain KpR24 using the LPS Extraction Kit (iNtRON Biotechnology, 17,141) according to the manufacturer’s protocol. Phage K2V3 (10^5^ PFU/mL) was preincubated with purified LPS at 37°C for 1 h. The mixture was then added to host cells, and adsorption was measured as described above. A control without LPS was included in parallel. All experiments were performed in triplicate.

### Virulence assessment using *galleria mellonella* model

*Galleria mellonella* larvae, obtained from Keyun Biotech, were acclimatized at 37°C for at least 24 h prior to infection. Larvae weighing between 300 and 400 mg were selected, surface-sanitized with 70% ethanol, and randomly distributed into sterile 90 mm Petri dishes, with 10 individuals per group. Bacterial cultures in the exponential phase were adjusted to an OD_600_ of 0.5 (approximately 10^8^ CFU/mL), washed three times with pre-cooled sterile PBS buffer, and pelleted by centrifugation. The bacterial pellet was resuspended in PBS buffer, and the cell concentration was verified using a light microscope and plate counting. A 5 μL aliquot of the bacterial suspension was injected into the last proleg of each larva, while a control group received an injection of 5 μL of sterile PBS buffer. Following injection, larvae were incubated at 37°C in the dark. Larval survival was monitored over a 6-day period; larvae unresponsive to touch and displaying melanization (blackening) were recorded as dead.

### Antibiotics susceptibility testing

Antibiotic susceptibility was assessed using the broth microdilution method in accordance with Clinical and Laboratory Standards Institute (CLSI) guidelines. Overnight cultures were adjusted to a 0.5 McFarland standard and subsequently diluted 100-fold in cation-adjusted Mueller-Hinton broth (CAMHB). A 50 μL aliquot of the diluted suspension was inoculated into wells of a 96-well plate containing 50 μL of CAMHB with serially diluted antibiotics. Wells containing sterile CAMHB served as blank controls. The plates were incubated at 37°C for 20 h. All experiments were performed in triplicate, and the median minimum inhibitory concentration (MIC) values were calculated.

### Bacterial competition assay

The quantitative competition assay was performed as previously described [29]. Briefly, overnight cultures were diluted 1:100 in fresh LB and incubated at 37°C until the OD_600_ reached 0.5. NUHL30457 and KpR24 were then mixed at a 1:1 initial ratio by adding 100 µL of each culture to 4 mL of fresh LB broth, and the mixture was co-incubated for 24 h. After competition, aliquots were serially diluted and plated on LB agar to obtain single colonies. To distinguish between the two strains, each single colony was picked and streaked onto double-layer agar plates supplemented with Kpph1. Growth on these selective plates served as the discriminator for strain identity. All single colonies derived from each LB plate were scored accordingly, and the colony count for each strain was recorded. The competitive index (CI) was then calculated as the output ratio of KpR24 to that of NUHL30457, normalized by their initial input ratio.

### Genome resequencing and analysis

Genomic DNA was extracted from Kpph1-resistant mutants using the FastPure Bacteria DNA Isolation Mini Kit (Vazyme, DC103-01), following the manufacturer’s instructions. DNA concentration and purity were assessed using a NanoDrop 2000 spectrophotometer. Subsequently, the samples were submitted to Meige Biotech Co. for whole-genome resequencing on the Illumina HiSeq PE150 platform.

### Fluctuation tests and mutation analysis

Fluctuation tests were conducted as previously described [30]. Briefly, overnight cultures were diluted 1:100 in fresh LB medium and incubated at 37°C until reaching an OD600 of 0.5. The cultures were then diluted 100-fold to a concentration of 10^3^ CFU/mL. Ten independent 1 mL aliquots (Group A) and one 10 mL bulk culture (Group B) were incubated at 37°C until the OD_600_ reached 0.6. For Group A, 100 μL from each tube was mixed with phage Kpph1 (an MOI of 100) and plated using the double-layer agar method. For Group B, ten 100 μL replicates were sampled from the single bulk tube for plating. Following 24 h of incubation on LB agar plates, phage-resistant colonies were enumerated. Mutation rates were calculated based on the total viable cell count and the number of resistant colonies. Finally, ten colonies were randomly selected from each plate, and the *wcaJ* gene was amplified and sequenced.

### Mutant construction and complementation

Gene knockout was performed using a CRISPR‑Cas9/λ‑Red recombination system as previously described [31]. The primers used for mutant construction are listed in Table S. A 20‑nt spacer targeting *wcaJ* was cloned into the pSGKP‑Km vector, and a synthetic 90‑nt ssDNA oligonucleotide with homology arms was employed as the repair template. The pCasKP-Hyg plasmid containing the Cas9 gene was first introduced into the wild-type strain by electroporation. After induction of the λ-Red recombination system with arabinose, the pSGKP-Km derivative and the ssDNA donor were co-transformed into the pCasKP-Hyg-harboring competent cells. Transformants were selected on hygromycin and kanamycin, and verified by PCR and DNA sequencing. To obtain marker-free mutants, the integration was selected on LB plates containing 5% sucrose at 37°C. For complementation, the whole sequence of *wcaJ* gene along with its native promoter regions were amplified by PCR and cloned into the pSTV28-Kan vector. The resulting plasmids were introduced into the mutant strains by electroporation, and the final constructs were verified by PCR and DNA sequencing.

### Transcriptome sequencing and data analysis

Briefly, 2 mL of exponentially growing bacterial cultures was collected and centrifuged at 10,000 × rpm for 2 min. The supernatant was removed and the precipitate was washed three times with 1× PBS buffer at 4°C. Total RNA was extracted using the RNeasy Mini Kit (Qiagen, 74,104) according to the manufacturer’s instructions. RNA integrity and concentration were assessed using a Qubit 3.0 Fluorometer and an Agilent 4200 system (Agilent Technologies). Ribosomal RNA was removed from the total RNA, and strand‑specific cDNA libraries were constructed using the ALFA‑SEQ RNA Library Prep Kit II (FINDROP, NRI002E), following the manufacturer’s protocol. The libraries were sequenced on an Illumina PE150 platform. Raw reads were processed with Fastp (v0.23.2) to obtain clean reads, and residual rRNA sequences were removed by alignment to the NCBI Rfam database using Bowtie2 (v2.4.5). The remaining reads were mapped to the reference genome with Bowtie2. Gene read counts were quantified using Salmon (v1.9.0), and normalized expression values (TPM) were calculated. Differential expression analysis between conditions was performed with DESeq2 (v1.34.0). Genes with an FDR ≤ 0.05 and an absolute log_2_(fold change) ≥1 were considered differentially expressed. GO and KEGG enrichment analyses of differentially expressed genes were carried out using clusterProfiler (v4.2.2).

### Statistical analysis

All experiments were performed with a minimum of three independent replicates. Data are expressed as means ± standard deviations and were analyzed using GraphPad Prism 8.4.3 software. Differences between groups were assessed using ordinary one-way analysis of variance (ANOVA) followed by Tukey’s post-hoc test. A *p* value ≤ 0.05 was considered statistically significant.

## Results

### Distinct phenotypic variants of phage-resistant CR-hvKP mutants

**Figure 1.**
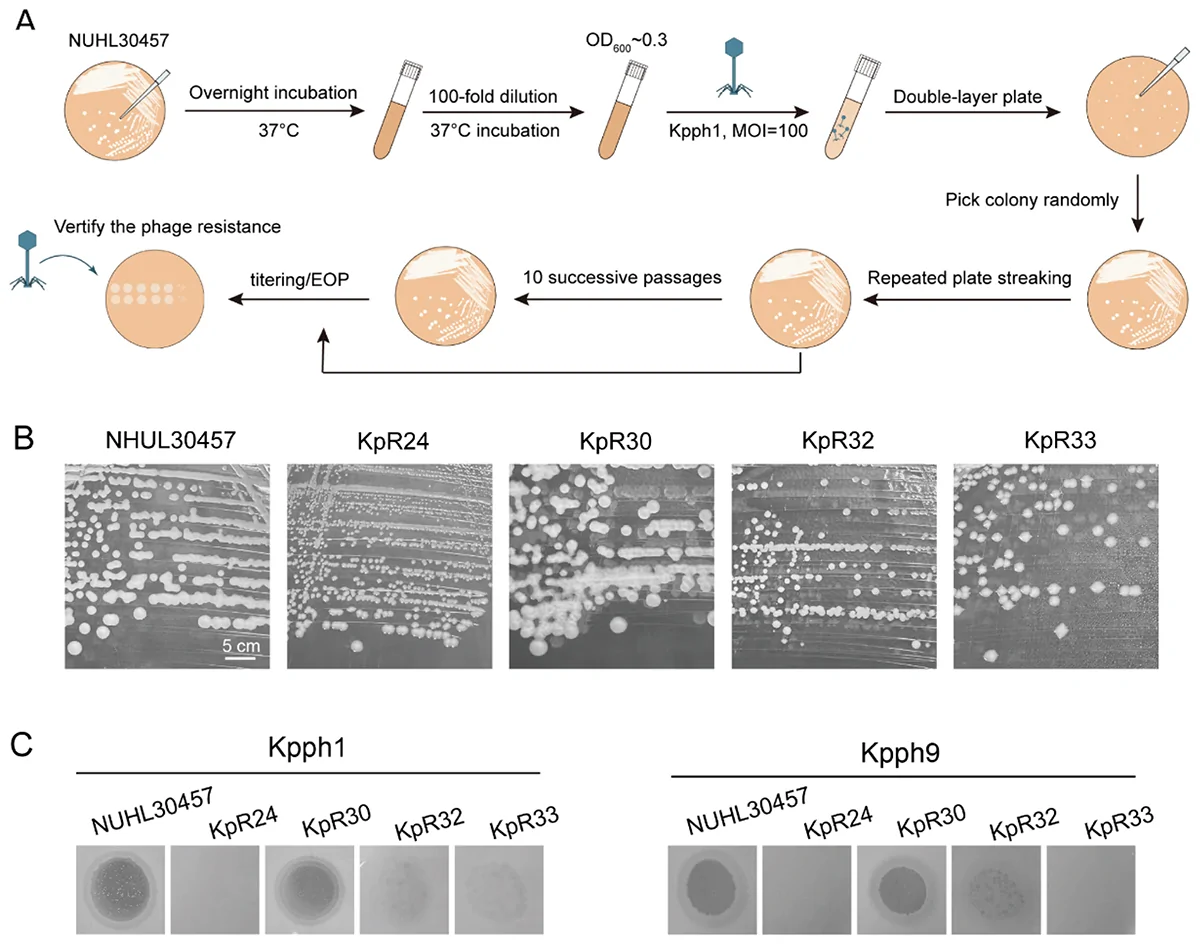
Isolation and characterization of phage-resistant mutants derived from *K. pneumoniae* strain NUHL30457. (A) Schematic overview of the procedure used to isolate and confirm phage-resistant mutants. (B) Colony morphology of the wild-type NUHL30457 strain and its derived phage-resistant mutants. (C) Plaque morphology produced by phage Kpph1 on lawns of the wild-type strain and resistant mutants.

Phage-resistant colonies emerged rapidly during the co-culture of NUHL30457 with phage Kpph1. Sixty colonies were randomly selected and subjected to three rounds of streaking to ensure purity. Plaque assays subsequently confirmed the stability of resistance (Figure 1(A)). Based on distinct morphological features and phage sensitivity profiles, the isolates were categorized into four phenotypic types for further characterization (Figure 1(B)). The predominant subset of isolates (55/60), represented by KpR24, formed small, rough, translucent colonies and exhibited complete resistance to Kpph1 (Figure 1(B,C)). The second type (3/60), exemplified by KpR30, displayed a hypermucoid phenotype while retaining sensitivity to phage infection. The third type, KpR32 (1/60), was characterized by tiny colony morphology and partial resistance. Conversely, the fourth type, KpR33 (1/60), formed transparent, rough colonies on which Kpph1 produced turbid, sparse plaques, indicative of a significantly reduced sensitivity. Furthermore, strains KpR24, KpR32, and KpR33 demonstrated cross-resistance to Kpph9, a member of the genus *Drulisvirus*. To extend this observation, we evaluated four additional lytic phages (Kpph2, Kpph5, Kpph6, and Kpph16) that vary from Kpph1 and Kpph9 in host range and plaque morphology (Supplementary Fig. S1A). Consistent with our original findings, these mutants exhibited markedly reduced susceptibility across this expanded panel (Supplementary Fig. S1B). This observation suggests that resistance mechanisms in these mutants are effective against phages across different taxonomic groups (Figure 1(C)).

### Confirming the resistance of mutants against phage

**Figure 2.**
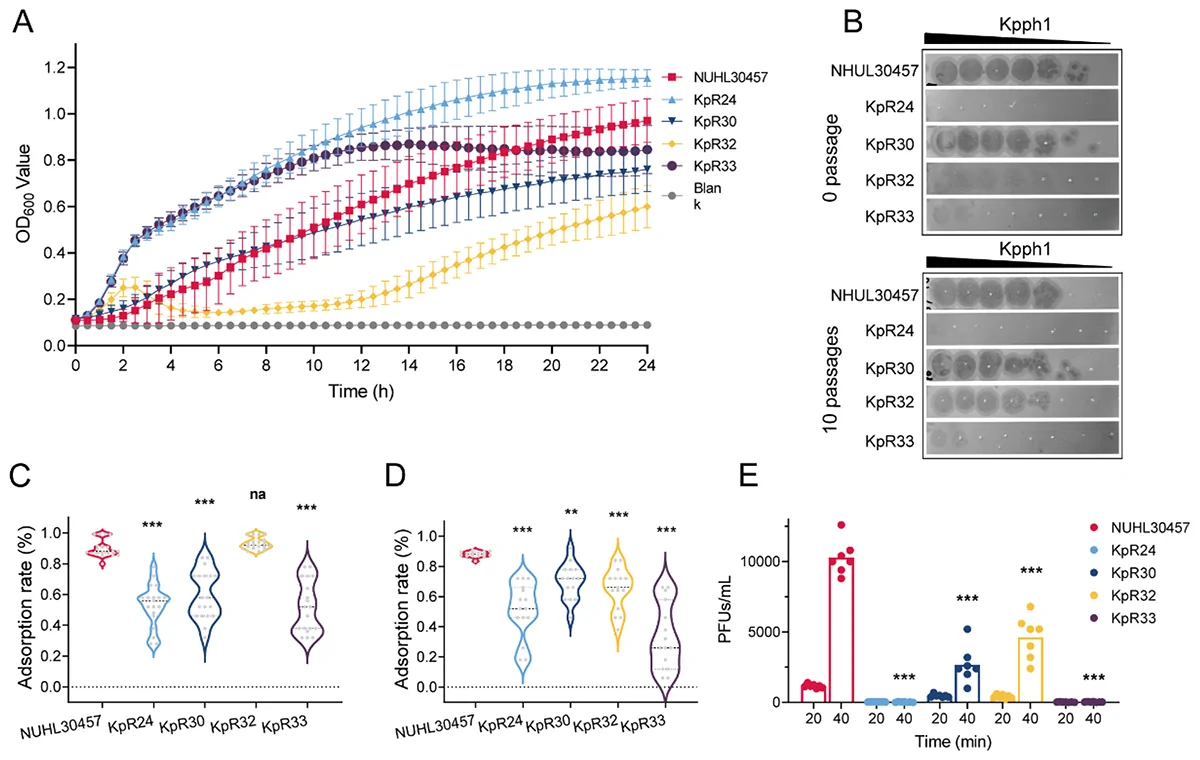
Characterization of phage resistance phenotypes in mutant strains. (A) Host killing curves of NUHL30457 and phage-resistant mutants following Kpph1 infection at an MOI of 100 (n = 20). (B) EOP of Kpph1 on wild-type and mutant strains before and after 10 serial passages. (C, D) Adsorption rate of Kpph1 to NUHL30457 and mutant host prior to (C, n = 19) and after 10 serial passages (D, n = 15). (E) Progeny phage titers of Kpph1 produced during co-cultivation with wild-type or resistant strains at 20 min and 40 min post-infection. Values are expressed as means ± the SD. Statistical significance was assessed using one-way ANOVA: *, *p* < 0.05; **, *p* < 0.01, ***, *p* < 0.001.

Host killing assays were conducted to quantitatively assess the degree of resistance in the mutants. An MOI of 100 was employed to recapitulate clinical high-dose phage therapy regimens and maximize the sensitivity of resistance detection [32]. Compared to the wild-type strain, all mutants exhibited varying degrees of resistance when co-cultured with phage Kpph1 (Figure 2(A)). Specifically, the growth of KpR24 remained unimpeded by phage exposure. KpR33 displayed a growth trajectory comparable to KpR24 during the initial eight-hour period; however, a subsequent gradual decline in optical density was observed, signifying the eventual onset of a productive phage infection. KpR30 and KpR32 demonstrated transient and mild resistance, which was confined to the first three and nine hours of co-culture, respectively. To further evaluate the stability of resistance to Kpph1, these mutant strains were subjected to 10 serial passages, followed by efficiency of plating (EOP) assays (Figure 2(B)). Consistent with the growth curves, KpR24 maintained complete, stable resistance to Kpph1, demonstrating no plaques observed across serial passages. The EOP of KpR33 was substantially lower than that of the wild-type strain and remained stable across passages. In contrast, KpR30 retained sensitivity to Kpph1, albeit with a marginally decreased EOP. Interestingly, although phage plaque formation on KpR32 was initially markedly reduced, phage susceptibility was restored following serial passaging.

To elucidate the specific barriers to infection, phage adsorption rates and replication kinetics were analyzed (Figure 2(C-E)). The adsorption rates of KpR24, KpR30, and KpR33 were significantly reduced relative to the wild-type strain, a pattern that persisted independent of serial passaging. In contrast, KpR32 initially exhibited adsorption kinetics comparable to those of the wild-type strain; however, this efficiency declined following serial passaging. Furthermore, phage replication efficiency was substantially diminished across all resistant mutants. Notably, no progeny phages production was detected in KpR24 and KpR33 after 40 min of co-culture. Collectively, these results suggest that the mutants employ distinct strategies to hinder phage adsorption and inhibit progeny production, thereby conferring resistance.

### Fitness cost of phage-resistant mutants

**Figure 3.**
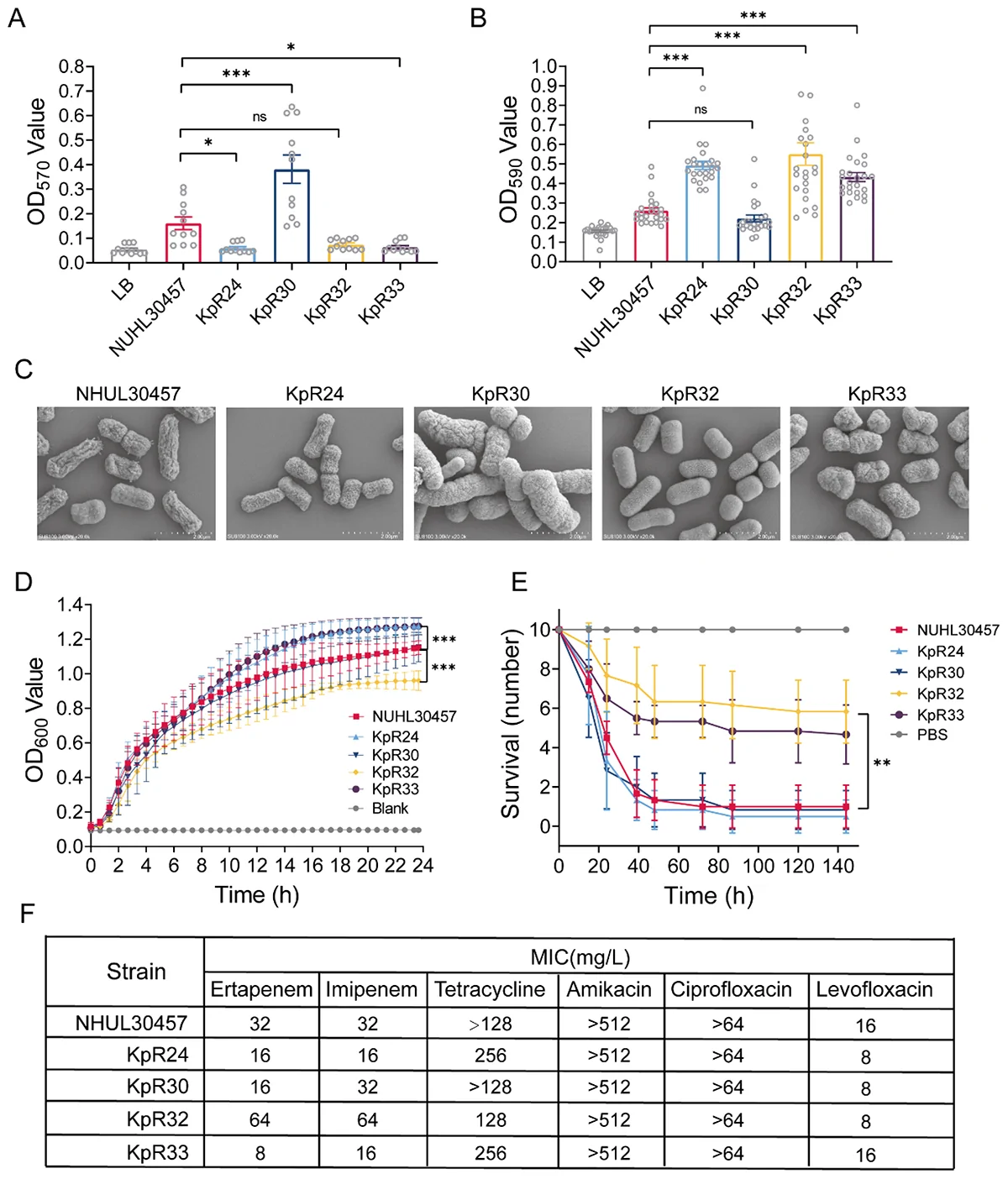
Assessment of fitness costs associated with phage-resistant mutants. Comparative analysis of NUHL30457 and phage-resistant mutants regarding (A) mucus viscosity (n = 11), (B) biofilm formation (n = 24), (C) cellular morphology observed via electron microscopy, (D) bacterial growth kinetics (n = 11), (E) virulence in a *Galleria mellonella* infection model (n = 6), and (F) antibiotic susceptibility (MIC) (n = 3). Data are presented as mean ± SD. Statistical significance was determined using one-way ANOVA: *, *p* < 0.05; **, *p* < 0.01, ***, *p* < 0.001.

To further investigate the mechanisms underlying resistance, capsular polysaccharide production and biofilm formation were quantified (Figure 3(A,B), and Supplementary Fig. S2). Relative to the wild-type strain NUHL30457, mutants KpR24, KpR32, and KpR33 produced significantly less capsular polysaccharide but exhibited enhanced biofilm formation. In contrast, KpR30 synthesized elevated levels of capsular polysaccharide than the wild type. These observations were corroborated by scanning electron microscopy (SEM), which revealed a prominent capsule surrounding KpR30, whereas the surface morphology of KpR24, KpR32, and KpR33 was markedly distinct from that of NUHL30457 (Figure 3(C)). The fitness costs associated with resistance were systematically evaluated using growth curves, *Galleria mellonella* virulence assays, and MIC determinations (Figure 3(D-F)). Among the mutants, KpR24 incurred a relatively low fitness cost, evidenced by a significantly increased growth rate relative to the wild type, while retaining comparable virulence. To evaluate the direct competitive fitness of KpR24, a competition assay was conducted against NUHL30457 (Supplementary Fig. S3). The results demonstrated that KpR24 exhibited marginally higher viable counts than NUHL30457, although the difference was not statistically significant (Supplementary Fig. S3A-B). The CI of KpR24 was 1.23, indicating that KpR24 possesses a slight competitive advantage over NUHL30457 under the tested conditions (Supplementary Fig. S3C). However, the MIC values of KpR24 for ertapenem, imipenem, and levofloxacin were decreased two-fold. KpR30 displayed growth kinetics and virulence comparable to those of the wild-type strain but showed heightened sensitivity to ertapenem and levofloxacin. In contrast, KpR32 exhibited significantly impaired growth kinetics, markedly attenuated virulence, and a two-fold increase in MIC values for both ertapenem and imipenem. Finally, KpR33 displayed a growth rate similar to that of KpR24 but presented reduced virulence and decreased susceptibility to ertapenem. Collectively, these findings confirm the phenotypic heterogeneity of phage-resistant mutants and highlight the variable fitness trade-offs imposed by resistance mechanisms.

### Identification of spontaneous mutations in phage-resistant mutants

**Figure 4.**
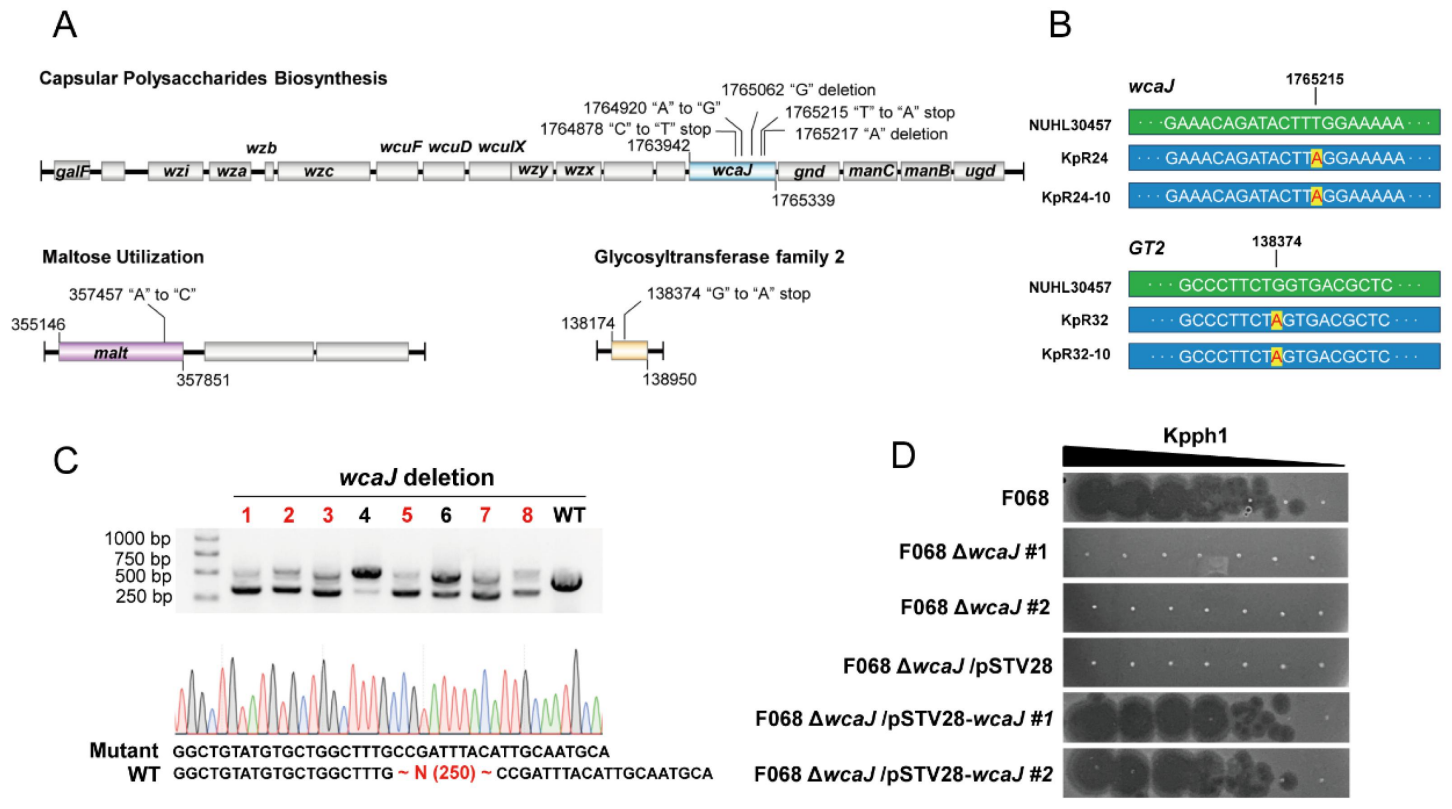
Genomic characterization and validation of phage resistance mechanisms. (A) Overview of genetic mutation sites identified in phage-resistant mutants. (B) PCR verification of the identified mutations. (C) Validation of the *wcaJ* gene knockout mutant. Representative cropped image; the full, uncropped parent image is supplied as underlying data (see Supplementary Fig S6). (D) EOP of phage Kpph1 against the wild-type F068, *wcaJ* knockout mutant, and complementary strains.

To identify the genetic determinants of phage resistance, whole-genome resequencing and comparative genomic analyses were performed using the wild-type strain NUHL30457 as a reference. To bolster the robustness of our findings, additional strains exhibiting phenotypes similar to KpR24 (KpR22, KpR34, KpR35, KpR36) and KpR30 (KpR29 and KpR31) were included (Supplementary Fig. S4A-D). All resistant mutants shared a single nucleotide mutation in *malT*, which encodes the transcriptional activator of the maltose regulon, at genomic position 357,457 bp (Figure 4(A)). Strains KpR24, KpR22, KpR34, KpR35, and KpR36 harbored mutations in *wcaJ*, a gene situated within the capsule production locus encoding undecaprenyl-phosphate glucose transferase (Figure 4(A)). Specifically, in KpR24, nucleotide alterations within the *wcaJ* open reading frames (ORFs) at genomic position 1,765,215 bp resulted in a premature stop codon via a TTG-to-TAG substitution (Figure 4(B)).

The *wcaJ* gene is essential for capsular polysaccharide biosynthesis. This identified mutation aligns with the significant impairment of capsule production observed in KpR24 (Figure 3(A)). Given that NUHL30457 exhibited resistance to high concentrations of kanamycin and hygromycin, which are the antibiotics required for the genetic manipulation system, the K2-type CR-hvKP strain F068 was selected as an alternative. To verify the role of *wcaJ* in phage sensitivity, *a wcaJ* knockout mutant was constructed in F068 background (Figure 4(C)). While wild-type F068 display sensitivity to Kpph1 with an efficiency of plating (EOP) comparable to that of NUHL30457, the *wcaJ*-deletion mutant exhibited no plaque formation (Figure 4(D)). Furthermore, complementation of the *wcaJ* mutant with plasmid pSTV28 expressing *wcaJ* restored phage susceptibility to levels comparable to wild-type F068, confirming the gene’s critical role in the phage infection process (Figure 4(D)).

In KpR32, a premature stop codon was introduced into the gene encoding a glycosyltransferase family 2 protein at genomic position 138,374 bp (Figure 4(A)). This mutation was stably maintained and remained detectable following 10 serial passages (Figure 4(B)). However, given that the EOP of Kpph1 on KpR32 recovered following passaging, resistance in this strain cannot be attributed solely to the glycosyltransferase mutation. Furthermore, no distinct mutations were identified in KpR30 or KpR33. Collectively, these findings underscore the genetic diversity and complexity of the resistance mechanisms employed by NUHL30457 against phage infection.

### Dominant phage-resistant mutants preexisted in the CR-hvKP communities

**Figure 5.**
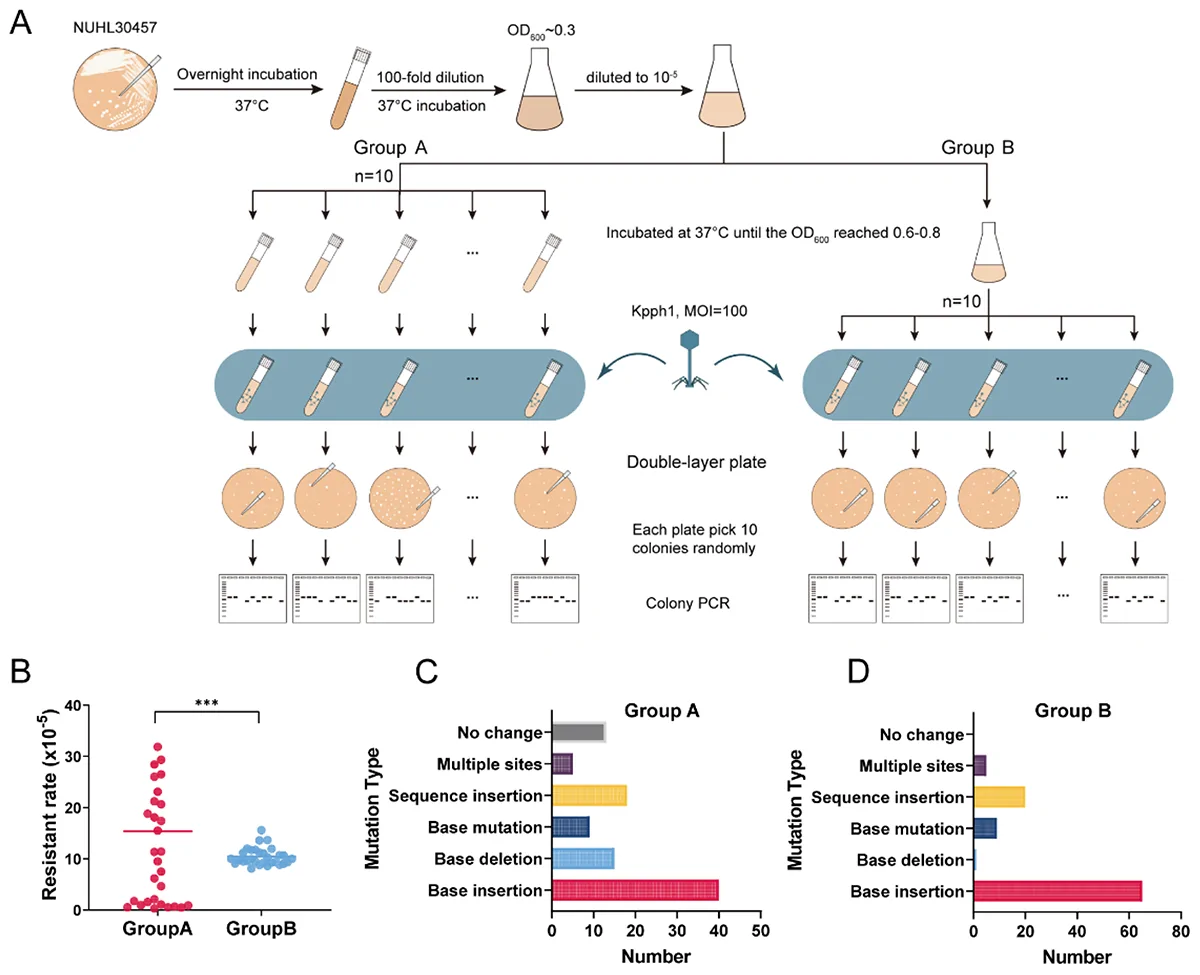
Fluctuation analysis and mutation profiling. (A) Schematic representation of the fluctuation test protocol and subsequent mutation analysis workflow. (B) Quantification of phage resistance rates in Group A versus Group B. (C and D) Classification and distribution of mutation types identified in Group A (C) and Group B (D).

To distinguish between selection-induced and preexisting resistance mechanisms, a fluctuation test was performed (Figure 5(A)). The number of resistant colonies exhibited significant variation between samples derived from independent populations (Group A) and those from the same population (Group B) (Figure 5(B)). Mutation rates in Group A ranged from 0.33 × 10^−5^ to 123.2 × 10^−5^, compared to a narrower range of 8.12 to 15.62 × 10^−5^ in Group B (Figure 5(B)). This significant variance indicates that phage-resistant mutants were present in the bacterial population prior to phage exposure. To assess the prevalence of *wcaJ* mutations within these preexisting resistant subpopulations, 100 mutants were randomly selected from Groups A and B for sequencing. Sequencing analysis revealed that 87% of mutants in Group A and 100% in Group B harbored mutations in the *wcaJ* ORF, identifying this locus as the predominant resistance mechanism under phage selection pressure (Figure 5(C,D) and Supplementary Table S1). Among *wcaJ* mutants, base insertion was the most frequent alteration (Figure 5(C,D) and Supplementary Table S1). Specifically, the insertion of an “AA” dinucleotide at position 1282 bp accounted for 40% of mutations in Group A and 65% in Group B. Insertion of the mobile element ISKpn26 (1088 bp) was the second most common type, representing 18% and 19% of the isolates in Groups A and B, respectively (Figure 5(C,D) and Supplementary Table S1). Less frequent mutation types included base deletions, single nucleotide substitutions, and multi-site mutations. Collectively, these findings demonstrate that *wcaJ* mutations represent the dominant mechanism of resistance and preexist within the population prior to phage selection.

### Transcriptomic insights into phage resistance

**Figure 6.**
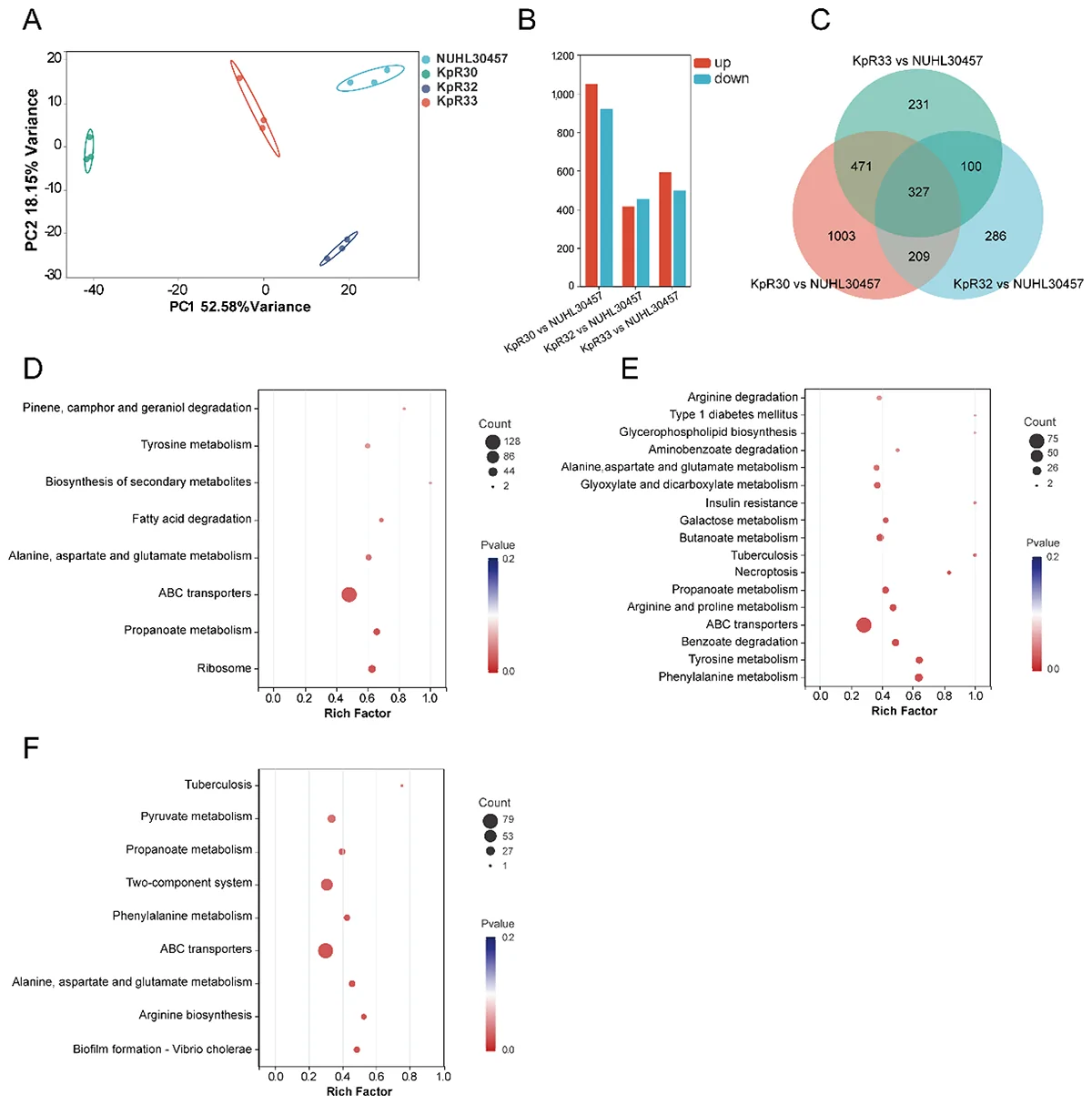
Comparative transcriptomic profiling of NUHL30457 and three phage-resistant strains (KpR30, KpR32, and KpR33). (A) PCA analysis illustrates the overall similarity and divergence of transcriptomic profiles among NUHL30457, KpR30, KpR32, and KpR33 strains. (B) Statistical summary of upregulated and downregulated DEGs in each phage-resistant mutant compared with NUHL30457. (C) Venn diagram showing the overlap and specificity of DEG sets identified from the three resistant strains. (D-F) KEGG functional enrichment analysis of DEGs derived from KpR30 (D), KpR32 (E), and KpR33 (F), respectively, versus NUHL30457 (n = 3).

To identify transcriptomic changes associated with phage resistance, we compared the gene expression profiles of the wild-type strain NUHL30457 and three phage-resistant mutants: KpR30, KpR32, and KpR33. Principal component analysis (PCA) separated the wild-type and resistant strains into distinct clusters, with the first two components explaining 52.58% and 18.15% of the total variance, respectively (Figure 6(A)). Each mutant displayed a large set of differentially expressed genes (DEGs) relative to the wild type. Venn diagram analysis revealed a core group of shared differentially expressed genes alongside gene sets unique to individual mutants, indicating conserved resistance mechanisms accompanied by strain-specific adaptations (Figure 6(B,C)).

Functional enrichment analysis showed that the differentially expressed genes were predominantly linked to membrane transport, amino acid metabolism, and cellular adaptation pathways, including ABC transporters, propanoate metabolism, and phenylalanine metabolism (Figure 6(D-F)). Beyond these shared enrichments, each mutant exhibited distinct pathway signatures. KpR30 exhibited specific enrichment for fatty acid degradation and ribosomal pathways, suggesting enhanced stress signal transduction in this mutant (Figure 6(D)). KpR32 showed enrichment for arginine and glycerophospholipid metabolism, indicating global metabolic remodeling to sustain fitness under phage pressure (Figure 6(E)). KpR33 displayed enrichment for two-component systems and biofilm formation pathways, pointing to a community resistance strategy against phage infection (Figure 6(F)). Together, these divergent strategies illustrate a multifactorial phage resistance model in CR-hvKP.

### Combination of Kpph1 and phage targeting Kpph1-resistant mutants

**Figure 7.**
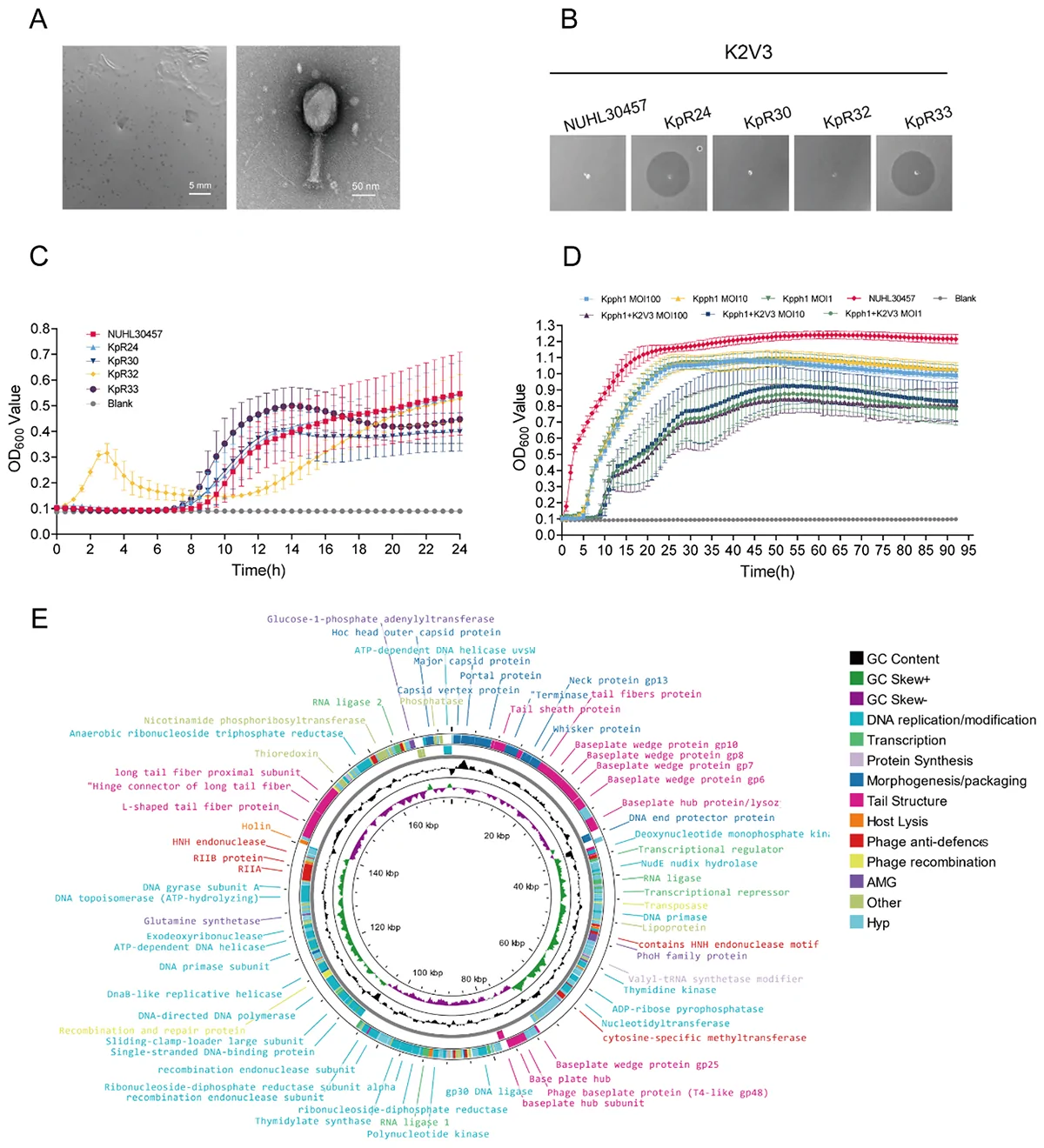
Characterization of the phage K2V3 against resistant mutants. (A) The morphology of phage K2V3. (B) plaques formation assays of K2V3 on NUHL30457 and phage-resistant mutants. (C) Host killing curves demonstrating the efficacy of combined treatment with phages Kpph1 and K2V3 (n = 20). (D) Long-term evolutionary dynamics of NUHL30457 under continuous induction by single Kpph1 and combined Kpph1 and K2V3 phage treatment (n = 20). (E) Genomic map of phage K2V3. Protein-coding sequences were predicted and annotated using RAST and Phanotate, with verification via BLASTp. The genome was visualized using Proksee.

To circumvent the resistance of dominant mutants to phage Kpph1, an alternative isolation strategy was employed using the resistant strain KpR24 as a host. This approach successfully yielded a novel phage, designated K2V3, which was subsequently purified. While K2V3 formed minute plaques on lawns of its isolation host, KpR24 (Figure 7(A)), it exhibited robust lytic activity against both KpR24 and KpR33, producing clear plaques indicative of effective infection (Figure 7(B)). To further explore the molecular basis underlying the phage adsorption of K2V3, we performed adsorption inhibition assays to identify potential host receptors (Supplementary Fig. S5). Given that K2V3 efficiently infects *wcaJ-*deficient mutants, other surface components besides CPS may contribute to phage adsorption. Proteinase K treatment of host cells only partially reduced K2V3 adsorption, suggesting that OMPs contribute to binding but are not the sole receptor (Supplementary Fig. S5A). To test whether LPS also plays a role, we incubated K2V3 with LPS extracted from KpR24 and then measured adsorption. This treatment did not significantly affect adsorption efficiency, indicating that LPS is not a major receptor for K2V3 (Supplementary Fig. S5B). Collectively, these results suggest that K2V3 utilizes multiple receptors, with OMPs representing one contributing component.

The dual-phage combination significantly suppressed the growth of NUHL30457 as well as all phage-resistant mutants (Figure 7(C)). The therapeutic potential of the phage combination Kpph1 and K2V3 was evaluated at MOIs of 1, 10, and 100 over a 94-hour period (Figure 7(D)). Relative to Kpph1 monotherapy, the co-administration of Kpph1 and K2V3 demonstrated significantly superior growth inhibitory activity, resulting in a prolonged bacterial lag phase and delaying the initiation of bacterial proliferation (Figure 7(D)). Notably, the combination therapy suppressed the proliferation of resistant mutants and mitigated the emergence of phage resistant mutants (Figure 7(D)). These results underscore the enhanced antibacterial efficacy and the capacity to inhibit resistance emergence.

Genomic analysis revealed that K2V3 possesses a linear double-stranded DNA genome of 174,330 bp and is classified within the genus *Slopekvirus* (family Straboviridae). It shares 97.4% nucleotide identity with *Klebsiella* phage KP5 and encodes 274 predicted ORFs, 108 of which are annotated as hypothetical proteins (Supplementary Table S2 and S3). The genome contains 24 structural tail proteins, including six associated with tail fibers, which may contribute to its host range (Figure 7(E) and Supplementary Table S3). For bacterial lysis, K2V3 encodes three holins, one spanin complex (comprising one O-spanin and one I-spanin), and one endolysin, forming an efficient lysis system (Figure 7(E) and Supplementary Table S2). Additionally, 13 genes are predicted to counter host defense systems, including an Anti-CBASS protein (Acb1), an HNH endonuclease, and a GhoS-like antitoxin associated with a type V toxin-antitoxin system (Figure 7(E) and Supplementary Table S2). Notably, the presences of a PhoH-like protein and a predicted inorganic phosphate transporter suggest a complex co-evolutionary relationship with its bacterial hosts.

## Discussion

Phage therapy has emerged as a promising alternative strategy for treating hypervirulent carbapenem-resistant bacterial infections. However, the development of bacterial resistance to phages remains a major obstacle to its broader clinical application. Understanding the resistance mechanisms of CR-hvKP can shed light on phage-bacteria interactions, thereby supporting the advancement of phage-based therapies [9]. In this study, we characterized phage-resistant CR-hvKP mutants with distinct morphological and infection phenotypes, analyzing their genetic changes and associated fitness costs. A dominant mutant type was identified, characterized by mutations in the *wcaJ* gene, which were predominantly present in the bacterial population prior to phage exposure. The inactivation of *wcaJ* resulted mainly from insertions of mobile elements, such as ISKpn26. This mutant type forms small, rough, translucent colonies, exhibits strong resistance to phage infection, and incurs minimal fitness costs. Additionally, partially resistant mutants were detected, indicating that CR-hvKP employs multiple defense strategies against phages and other environmental stresses. These findings highlight the complexity of bacterial resistance mechanisms and underscore the need for further investigation into optimizing phage therapy approaches.

Mutations in genes responsible for capsule production are well-documented mechanisms of phage resistance in *K. pneumoniae* [30,33,34]. Specifically, the *wcaJ* gene encodes a glycosyltransferase that initiates the production of CPS by transferring glucose-1-phosphate to undecaprenyl phosphate, forming undecaprenyl pyrophosphoryl-glucose (Und-PP-Glu), a key intermediate in capsule production [35]. Consequently, *wcaJ* activity directly regulates extracellular capsule production and the generation of metabolic intermediates. Prior work has shown that disrupting *wcaJ* alters the synthesis of colanic acid, a predominant exopolysaccharide in Enterobacteriaceae, thereby influencing mucoid phenotypes and contributing to phage resistance [36]. Beyond its role in capsule production, loss-of-function mutations in *wcaJ* have been linked to increased resistance to complement-mediated killing, complement binding, and opsonophagocytosis, underscoring its broader importance in the survival and evolution of *K. pneumoniae* within a host [37]. Consistent with previous findings, our results confirm that *wcaJ* is a critical target for bacterial adaptation under phage pressure. We found that various genetic disruptions, including insertions, deletions, and substitutions, can effectively halt capsule production, thereby preventing phage infection. This capacity to modulate defense mechanisms via a single gene represents a key evolutionary strategy, enabling *K. pneumoniae* to rapidly adapt to hostile environments. The role of mobile genetic elements in this process is also particularly noteworthy. Insertion sequences (IS) that interrupt CPS biosynthesis genes have been previously identified as a primary driver of resistance in carbapenem-resistant *K. pneumoniae* [38]. By interrupting these genes, bacteria can reversibly switch capsule production “on” or “off,” a strategy that enables rapid adaptation to environmental stress [34]. Moreover, the insertion of these elements can modulate the conjugation frequency of plasmids carrying resistance and/or virulence factors, thereby shaping resistance and virulence profiles [39]. Consequently, by regulating *wcaJ* through mutation or IS element insertion, the bacterium can fine-tune its response to environmental stress, optimizing its survival and colonization potential. It is important to note that while *wcaJ* mutations did not completely block phage adsorption, the release of progeny phages was fully inhibited, a finding consistent with prior research [34]. This observation implies that the mutation may interfere with binding to secondary receptors, thereby preventing the successful injection of phage DNA [18]. Further studies are required to fully elucidate the precise details of this resistance mechanism.

Bacteria utilize diverse innate defense systems, such as CRISPR-Cas, restriction-modification (RM), and abortive infection mechanisms, to counteract phage threats [16]. Notably, CRISPR-Cas systems are highly prevalent in clinical *K. pneumoniae* isolates, with one study reporting their presence in 41.2% of genomes [40]. Among these, Type I-E, subtype I-E*, and Type I-F are the primary systems identified. In recent years, numerous additional anti-phage systems have been discovered in this species [17]. Nevertheless, direct evidence supporting the functionality of these systems remains limited [17]. It is noteworthy that receptor blocking is a predominant mechanism observed during the short-term co-culture of *K. pneumoniae* with its phages, implying the efficacy of this rapid response strategy against phage infection. However, the role of this mechanism in long-term phage-bacterium co-evolution requires further investigation. Beyond the disruption of phage adsorption, *K. pneumoniae* employs various other defensive tactics, including delaying phage development and altering mutation rates [41,42]. Our resistance profiling revealed the consistent identification of mutations in the *malT* gene across all resistant isolates. Previous studies have demonstrated that the knockdown of *malT* contributes to reduced susceptibility of *E. coli* to phage infection via the downregulation of phage receptors [43]. Although these alterations suggest a potential role in bacterial defense, *malT* inactivation is insufficient to account for the observed phenotypic variation on its own. Rather, mutations in *malT* may constitute a baseline defense mechanism, modulating cellular physiology to render the host environment refractory to phage infection. However, the magnitude of phage resistance may be modulated by regulatory effects at the transcriptional or translational level. In this study, we characterized three additional resistant strains that exhibit distinct phenotypic shifts, although genome resequencing did not reveal specific genetic mutations responsible for their resistance, whereas transcriptomic analysis provided critical insights. Strain KpR30 displayed a hypermucoid phenotype associated with reduced phage adsorption and replication. This strain exhibited the coordinated upregulation of fatty acid degradation and ribosomal components, which suggests a resistance mechanism predicated primarily on receptor masking. In contrast, KpR32 demonstrated significant growth inhibition, manifesting as smaller colonies and a reduced proliferation rate compared to the wild-type strain. Furthermore, KpR32 displayed metabolic re-routing, characterized by upregulated arginine and glycerophospholipid metabolism. These findings imply a shift in cellular energetics and membrane composition potentially interfering with phage replication or progeny release. Concurrently, phage EOP and adsorption rates in KpR33 were significantly altered, although infection capability was partially restored after a period of co-culture. Transcriptomic analysis revealed the activation of two‑component systems and biofilm‑associated genes, indicating the potential involvement of cell density-dependent regulation. Collectively, these findings highlight the multifaceted complexity of phage resistance in *K. pneumoniae*. The emergence of resistant mutants with clear phenotypic changes, yet lacking identifiable genetic markers, suggests that significant aspects of these defense mechanisms are likely orchestrated by transcriptional reprogramming. Further investigations are warranted to fully elucidate the varied strategies *K. pneumoniae* employs to withstand phage pressure.

The restoration of antibiotic susceptibility is a significant fitness cost often associated with phage resistance, providing a strong theoretical basis for combining phages with antibiotics [13,44–46]. Typically, a trade-off exists where resistance to phages compromises a bacterium’s resistance to antibiotics. This phenomenon can arise through phage-induced deletions of antibiotic resistance genes [47]. For instance, infection by phage KP36 selects for deletions encompassing the *bla*CTX-M, *ant*(3’’), *sul*2, *folA*, and *mph(E)*/*mph(G)* resistance clusters, resulting in phage-resistant clones that exhibit an immediate switch to full drug susceptibility [46]. Alternatively, the trade-off may exist because the surface structures targeted by phages also play a role in antibiotic efflux or entry [13,48]. This phenomenon is known as pleiotropy, an evolutionary effect where the evolution of a single bacterial gene impacts multiple traits [49]. For example, mutations in the outer-membrane porin OprM (a phage adsorption receptor) can confer phage resistance. OprM is also a component of multidrug efflux systems (affecting ceftazidime, ciprofloxacin, tetracycline, and erythromycin). Consequently, alterations in OprM significantly enhance susceptibility to these antibiotics [50]. However, this relationship is not uniform. Previous research has also reported that mutations, particularly those affecting LPS synthesis, have been shown to enhance resistance to certain antibiotics, highlighting the complex evolutionary interplay between phage and antibiotic resistance [51]. In the present study, we observed that the phage-resistant mutant KpR32 exhibited increased resistance to carbapenem antibiotics, despite lacking significant genomic alterations. Unlike the typical genetic trade-off, KpR32 appeared to enter a state of metabolic inhibition upon exposure to phage Kpph1. This physiological slowdown likely constitutes a defensive mechanism wherein reduced metabolic activity impedes phage replication and progeny release. Strains capable of resisting both phages and antibiotics through such physiological adaptations pose a potential risk of persisting during combination therapies. Consequently, vigilant monitoring of these resilient pathogens is essential [30]. Further multi-omics investigations, including transcriptomics and metabolomics, are warranted to fully elucidate the mechanisms underlying this dual-resistance phenotype.

Phage cocktails, which combine two or more phages, are widely used to overcome the limitations of single-phage therapy [52]. In animal models of *K. pneumoniae* burn wound infections, cocktail therapy has proven more effective than monotherapy in reducing bacterial burden and suppressing the emergence of resistant strains [53]. By targeting distinct bacterial receptors, these cocktails offer broader coverage and more potent inhibition. However, designing effective cocktails remains challenging due to the complex interactions between phages and their bacterial hosts [54]. A practical approach to optimization involves screening bacteria that have developed resistance to the initial phage and identifying new phages capable of lysing both the original strain and these resistant mutants. This method allows for the formulation of tailored cocktails that specifically counteract emerging phage-resistant subpopulations [55]. Such formulations have been shown to significantly lower the frequency of resistance and improve therapeutic outcomes in mouse models compared to single phages [56]. Furthermore, recent strategies exploiting collateral sensitivity have been successfully used to construct effective phage cocktails. This strategy alleviated bacterial burden and suppressed phage resistance in CR-hvKP, highlighting the importance of understanding resistance mechanisms in the design of phage therapies [57]. Although adsorption inhibition assays preliminarily confirmed that OMPs contribute to K2V3 adsorption and support a multi-faceted receptor mode, the specific functional receptor molecules, key structural domains, and their individual contributions to phage binding remain unclear. Future research is required to clarify the host range specificity and provide theoretical support for practical phage application. Furthermore, while our results demonstrate robust in vitro suppression of resistance, validating these findings in in vivo models remains a critical next step to confirm the therapeutic potential of the phage cocktail under complex physiological conditions.

## Conclusion

In conclusion, this study illuminates the adaptive strategies CR-hvKP employs to evade phage predation. We identified *wcaJ* mutation as the predominant resistance mechanism, offering effective protection with minimal fitness cost. Additionally, the observation of four distinct resistance phenotypes underscores the multifaceted nature of bacterial defense systems in response to phage pressure. Furthermore, the demonstrated efficacy of the phage cocktail comprising Kpph1 and K2V3 highlights the potential of rationally designed combinations to suppress the emergence of resistant mutants. Collectively, these findings provide a critical framework for understanding phage-bacteria dynamics and offer a strategic rationale for optimizing clinical phage therapies against multidrug-resistant bacterial pathogens.

## Supplementary Material

Supplementary_Table S1.xlsx

Supplementary_Table S3.xlsx

Supplementary_Table S2.xlsx

266627912.R1_ Disclosure_Form-2026.docx

CleanSupplementary_Files_v5.docx

## Data Availability

The genome sequence data for phages K2V3 has been submitted to the National Microbiology Data Center (NMDC) under accession number NMDC60227343 (https://nmdc.cn/resource/genomics/genome/detail/NMDC60227343) . The raw transcriptomic data have been deposited in the NCBI Sequence Read Archive (SRA) under accession number PRJNA1501910 The data used to generate the figures in this study have been uploaded to Zenodo (https://doi.org/10.5281/zenodo.22039500) .

## Open scholarship

This article has earned the Center for Open Science badge for Open Data. The data are openly accessible at https://nmdc.cn/resource/genomics/genome/detail/NMDC60227343; https://www.ncbi.nlm.nih.gov/sra/?term=PRJNA1501910; https://doi.org/10.5281/zenodo.22039500
