# Supplementary material for: Phenotypic heterogeneity and genetic mechanisms of phage resistance in hypervirulent carbapenem-resistant Klebsiella pneumoniae
Source: Virulence. 2026 Sep 9;17(1):2732318. doi: 10.1080/21505594.2026.2732318 (PMC13577281; doi:10.1080/21505594.2026.2732318)
Supplement: Supplementary file 1 [file KVIR_A_2732318_SM3300.docx]

# Phenotypic Heterogeneity and Genetic Mechanisms of Phage Resistance in Hypervirulent Carbapenem-Resistant *Klebsiella pneumoniae*

Ye Huang^1,2^*^#^, Yichen Liu^1,3^*, Shimin Chen^1,3^, Ziyue Fan^1,4^, Lili Yan^1,2^, Fanglin Zheng^1,2^, Yang Liu^2^, Xinping Xu^1,2#^

^1^ Jiangxi Provincial Key Laboratory of Respiratory Diseases, Jiangxi Institute of Respiratory Diseases, The Department of Respiratory and Critical Care Medicine, The First Affiliated Hospital, Jiangxi Medical College, Nanchang University, Nanchang, Jiangxi 330006, P.R. China.

^2^ China-Japan Friendship Jiangxi Hospital, National Regional Center for Respiratory Medicine, Nanchang, Jiangxi 330200, P.R. China

^3^ Gerontology Department of The First Affiliated Hospital, Jiangxi Medical College, Nanchang University, Nanchang, Jiangxi 330052, P.R. China

^4^ Queen Mary College, Nanchang University, Nanchang, Jiangxi 330031, P.R. China

**^#^ Correspondence:**

Ye Huang

ndyfy09775@ncu.edu.cn

Xinping Xu

xinpingxu@ncu.edu.cn

*** Equally contributed to this work.**

Supplementary Fig S1 Plaque morphology produced by phages targeting NUHL30457 on lawns of the wild-type strain (A) and resistant mutants (B).

Supplementary Fig S2 Quantitative analysis of capsular polysaccharides of NUHL30457 and phage-resistant mutants.

Supplementary Fig S3 Competition assay between strain NUHL30457 and the resistant strain KpR24. (A) Screening and differentiation method for co-cultured NUHL30457 and KpR24 strains. Single colonies obtained by dilution plating were streaked on double-layer plates containing phage Kpph1, and colonies capable of growth were identified as the phage-resistant strain KpR24. (B) Cell counts of NUHL30457 and KpR24 in the co-culture system at 0 h and 24 h of incubation (n = 20). (C) The competitive index of KpR24 relative to NUHL30457, reflecting the relative competitive fitness of the resistant strain during co-cultivation (n = 20). Statistical analysis was performed using the Mann-Whitney test, and a *P* value < 0.05 was considered statistically significant.

 Supplementary Fig S4 Plaque morphology and fitness costs assessment of phage-resistant mutants. (A) Plaque morphology produced by phage Kpph1 and Kpph9 on lawns of the wild-type strain and resistant mutants. Comparative analysis of NUHL30457 and phage-resistant mutants regarding (B) mucus viscosity (n = 11), (C) biofilm formation (n = 24), (D) bacterial growth kinetics (n = 11).

Supplementary Fig S5 Receptor recognition assays for phage K2V3. (A) Adsorption efficiency of K2V3 following proteinase K treatment. PBS served as the control. (B) Adsorption efficiency of K2V3 in the presence of LPS. Tris‑HCl served as the control. Statistical analysis was performed using the Mann-Whitney test, and a *P* value < 0.05 was considered statistically significant.

2000 bp

2500 bp

3000 bp

4000 bp

5000 bp

6000 bp

8000 bp

10000 bp

1500 bp

1000 bp

750 bp

500 bp

250 bp

Supplementary Fig S6 The full, uncropped parent image of Figure 4C.
